# Estimation of a Neonatal Health Production Function for Iran: Secondary Analysis of Iran’s Multiple Indicator Demographic and Health Survey 2010

**Published:** 2019-08

**Authors:** Mostafa AMINI-RARANI, Arash RASHIDIAN, Mohsen BAYATI, Esmaeil KHEDMATI MORASAE

**Affiliations:** 1. Social Determinants of Health Research Center, Isfahan University of Medical Sciences, Isfahan, Iran; 2. Department of Health Management and Economics, School of Public Health, Tehran University of Medical Sciences, Tehran, Iran; 3. Department of Information, Evidence and Research, World Health Organization, Eastern Mediterranean Region, Cairo, Egypt; 4. Health Human Resources Research Center, School of Management & Information Sciences, Shiraz University of Medical Sciences, Shiraz, Iran; 5. Center for Systems Studies, Hull University Business School (HUBS), Hull York Medical School (HYMS), University of Hull, Hull, UK

**Keywords:** Neonatal mortality, Health production function, Iran

## Abstract

**Background:**

Despite constant decrease in rate of neonatal mortality, the rate is still higher than that of other under-five children. One of the first steps towards reduction of neonatal mortality is to identify its determinants using health production function. The aim of the present study was to estimate neonatal health production function for Iran.

**Methods:**

In this cross-sectional study, Iranian Multiple Indicator Demographic and Health Survey (Ir-MIDHS) 2010 was used. Four categories of socioeconomic, mother, neonatal demographic and healthcare system factors were entered into the Binomial Logistic Regression model to estimate neonate health production function. Households’ economic status was constructed using principal component analysis.

**Results:**

History of abortion/stillbirth had the highest significant positive impact on odds of neonatal mortality (odds ratio=1.98; 95 % CI=1.55–2.75), indicating that neonates of mothers with such a history had 1.98 times higher chance of death compared to other neonates. Moreover, odds ratio of neonatal death for the poorest quintiles was 1.70 (95 % CI=1.08–2.74), indicating that by moving from the poorest quintile to the richest one, the odds of being alive for neonates increased up to 70%. However, skilled birth attendant decreased the chance of death up to 58% (odds ratio=0.58; 95 % CI=0.36–0.93).

**Conclusion:**

Considering the most significant inputs of neonatal health production function in Iran, improvement of economic status of households, provision of appropriate care services for mothers, and improvement of delivery care provided by trained personnel, could be priorities for health policymakers to act and reduce neonatal mortality in Iran.

## Introduction

Health promotion, as one of the main aims of healthcare systems, is of salient importance for neonate (1). Consequently, neonatal mortality rates have been used (2), along with other indices of mortality and life expectancy at birth, to evaluate societies’ health status (3). Not only neonatal mortality rates considered as health outcomes, but also they reflect societies’ socioeconomic development level (4, 5). Neonatal mortality (death during first 28 d after birth), among other child mortality indices, is of special significance as most of under-five (more than 60%), and infant (more 70%) happens during neonate period (6). Moreover, illness in neonate period imposes remarkable costs over healthcare, education, and public supportive systems in future (7). Despite falling rates and levels of neonatal mortality (8), declines in the neonatal mortality rate are slower than mortality among children aged 1–59 months. Moreover, regardless of scaling up the relevant child health policies around the globe, the inequality in child mortalities are still notable between and among countries (9). According to levels and trends in child mortality report 2017, obvious disparities in neonatal mortality exist across regions and countries. Among regions, neonatal mortality was highest in sub-Saharan Africa and Southern Asia up to 28 deaths per 1000 live births, and across countries, neonatal mortality rates ranged from 46 deaths (per 1,000 live births) in Pakistan to 1 each in Iceland and Japan (8).

In Iran, neonatal mortality rates show that substantial progress has been made in reducing neonatal deaths per 1000 live births from 25 to 10 since 1990 to 2016 (8). However, in line with achieving Sustainable Development Goal 3 and 10 (SDGs 3 and 10) by 2030, there is still much to hop and do for more reduction in the neonatal mortality rate (6) as well as reduction in the neonatal mortality inequality (10). The most important step towards reduction of neonatal mortality is to identify its determinants. Having that done, relevant interventions can be tailored for before, during, and after delivery that help reduce neonatal mortality (11).

There are different factors that influence neonatal mortality. Basically, neonatal mortality level is related to socioeconomic conditions of society (12). These factors, usually, affect neonatal health through their influence on mothers’ health. Factors like demographic features of mother and neonate (13), delivery type and location, physical conditions of living, mother’s health status (11), provision of pre-pregnancy and delivery care by skilled personnel (14), stillbirth history (15), mother and father education level and their occupation (16) are of factors reported as determinants of neonatal mortality.

Health determinants are usually analyzed within a framework called health production function in which determinants are considered as inputs and health status is considered as outcome of function (17).

The paper’s major objectives were to estimate neonatal health production function and to identify the main factors affecting neonatal death in Iran using data from Multiple Indicator Demographic and Health Survey (MIDHS) 2010. The results are expected to inform policymakers about neonatal death determinants and as a result to provide invaluable information to the child health policy-making process.

## Methods

### Data

In this cross-sectional secondary data analysis, MIDHS data for 2010 was used to conduct the study. To gather required data, multistage stratified random cluster sampling method was used in MIDHS to gather data. The minimal number of samples from each province was estimated to be 400 households. Distribution of samples across the country was based on households’ number in each province in Iran and, finally, 31300 households were selected into the survey (18).

### Model and variable

Considering relevant literature, neonatal mortality determinants were grouped into 4 categories of socioeconomic (SES), mother (M), neonatal demographic (D), and healthcare system factors (HS). Accordingly, the preliminary model will be as follows:

ND=f(SES,M,D,HS)

In other words, neonatal death (ND) is a function of those four factors. Taking data availability and using a well-known framework (19), household economic status (HES) and location of residence (L); mother education level (MEL) and history of abortion /stillbirth (HAS); neonate’s sex (S); and skilled prenatal care (SPC) and skilled birth attendant (SBA) were entered into the model from socioeconomic, mother, neonatal demographic, and healthcare system categories, respectively. The model structure, particularly variables selection was built according to the latest literature on neonatal mortality/ health (12, 15, 20, 21).

ND=f(HES,MEL,L,S,SPC,SBA,HAS)

### Variables definition

Neonatal death, as our dependent variable, was chosen as a dichotomous outcome, i.e. whether each of the mother’s alive birth was still alive or already died (death during the first 29 days after birth). As yearly estimates of mortality are not accurate enough (due to relative scarcity of death numbers) (22), and also as sufficient numbers of births reduces sampling error effects (10), a 10-year long period was used for live birth estimates. Eventually, 33144 live births from 2000 to 2010 were entered into the model.

Using principal component analysis (PCA) (23) and data from 30870 households in 2010, households’ economic status was measured. Consequently, economic quintiles of “poorest”, “poor”, “middle”, “rich”, and “richest” were constructed and entered, as a categorical variable, into regression model. Mother education level was also considered as a categorical variable including illiterate, primary, guidance, high school, pre-academic, and academic categories.

By skilled prenatal care, it was meant any care provided by specialists, General Physicians (GPs), trained midwives, and family health experts. Moreover, by skilled birth attendant, it was meant childbirth attended by specialists, GPs, midwives, trained personnel, and multipurpose health workers (Behvarz).

Abortion was defined as pregnancy finished before 20-week of pregnancy. Stillbirth, in contrast, was defined as pregnancy that spanned more than 20-week but led to a neonate birth that had no vital signs after delivery.

### Data analysis

Descriptive statistics were used to describe variables. In the present study, as dependent variable was binomial (neonates alive or not) and several independent variables were included in the model, the multivariate binomial logistic regression (adjusted logistic regression) was used to estimate neonate health production function. Furthermore, it was tried to inter variables that may have potential effects on neonatal death in model as independent variables to control their effects.

All analyses were done by in STATA/SE (version 14; Stata Corporation, College Station, TX, USA). The MIDHS stratification, cluster sampling, and unequal selection probabilities were considered in the analyses via *svyset* command (23). As commonly used traditional goodness-of-fit tests like Hosmer–Lemeshow and Pearson’s chi-square goodness-of-fit are subjected to some problems associated with logistic regression model using survey data, in this study we applied F-adjusted mean residual test to assess logistic regression goodness-of-fit applying *svylogitgof* command (24).

## Results

Table 1 illustrates a descriptive scheme of neonatal mortality and its determinants in Iran in 2010. Of 33144 live births studied from 2000 to 2010, 329 ones (around 1%) had died. In terms of socioeconomic status, 22.4% of families belonged to the poorest quintile. Around 10% of mothers were illiterate and 15% had academic level education. Most of the households were living in urban areas (66%). 52.4% of neonates were male. Most mothers (83%) had enjoyed from pregnancy services, care, and 82% given childbirth with help of a skilled attendant. Table 2 shows the regression analysis results. History of abortion or stillbirth, household’s economic status, and neonate’s sex had, respectively, the highest significant positive association on odds of neonatal mortality.

Moreover, skilled attendant childbirth had the highest significant negative effect on odds of neonatal death.

**Table 1:** Descriptive statistics of variables

***Variables***	***Frequency***	***Percent***
Neonatal death	329	0.99
Household economic status		
Poorest	7114	21.46
Poorer	6947	20.97
Middle	6597	19.90
Richer	6304	19.02
Richest	6182	18.65
Sum	33144	100
Mother’s education level		
Illiterate	3400	10.25
Primary/literacy movement	7526	22.71
Secondary school	4825	14.56
High school	5609	16.92
Pre-university	6621	19.98
University	5163	15.58
Sum	33144	100
Location of residence		
Urban	21956	66.24
Rural	11188	33.76
Sum	33144	100
neonate sex		
Male	17367	52.37
Female	15777	47.63
Sum	33144	100
Using skilled prenatal care	27379	82.61
Skilled birth attendance	27080	81.70
History of abortion/stillbirth	4223	18.59

**Table 2:** Estimates of neonatal health production function in Iran (2010), logit model

***Variable***	***Coefficient***	***Adjusted Odds ratio***	***P-value***	***95% CI***	
				***Lower***	***Upper***
Household economic status					
Poorest	0.49	1.70	0.05	1.08	2.74
Poorer	0.53	1.63	0.04	1.00	2.89
Middle	−0.08	0.92	0.77	0.52	1.60
Richer	−0.01	0.97	0.95	0.59	1.73
Richest^*^	1	1	-	-	-
Mother’s education level					
Illiterate	0.18	1.20	0.56	0.63	2.26
Primary/literacy movement	−0.31	0.73	0.28	0.41	1.30
Secondary school	−0.15	0.86	0.62	0.47	1.56
High school	0.33	1.39	0.27	0.78	2.52
Pre-university	−0.30	0.74	0.33	0.40	1.36
University^*^	1	1	-	-	-
Location of residence					
Rural	0.21	1.23	0.15	0.91	1.67
Urban^*^	1	1	-	-	-
neonate sex					
Male	0.30	1.35	0.01	1.05	1.72
Female^*^	1	1	-	-	-
Skilled prenatal care	−0.24	0.78	0.35	0.47	1.29
Skilled birth attendance	−0.54	0.58	0.02	0.36	0.93
History of abortion/ stillbirth	0.73	1.98	<0.001	1.55	2.75
Constant	−4.24	0.014	<0.001	0.01	0.03

* denote reference group

Positive impact abortion or stillbirth history on neonatal death means that neonates of mothers with such a history had 1.98 times higher chance of death compared to other neonates. Across economic quintiles, the poorest and poor quintiles had a positive association with neonatal mortality. According to Table 2, odds ratio of neonatal death for the poorest quintile was 1.70, indicating that by moving from the poorest quintile to the richest one, the odds of being alive for neonates increased 70%. For the poor quintile, the odds ratio was 1.63, meaning that, compared with the richest one, neonates in that quintile had 63% higher chance to lose their life over their first 29 d of life. In terms of sex, being male increased the chance of death 35% (odds ratio=1.35), compared with their female counterparts.

In terms of negative impact on odds of neonatal death, skilled birth attendant decreased the chance of death around 58%. All other variables including mother education, location of residence, and skilled prenatal care had no significant effect on neonatal death.

Finally, after fitting the logistic regression model considering the survey sampling design, the F-adjusted mean residual goodness-of-fit test was done and proposed no evidence of lack of fit (F-adjusted test statistic = F (9,11831), Prob> F= 0.12).

## Discussion

The present study aimed to estimate neonatal health production function in Iran. In overall, of socioeconomic, mother-related, neonatal demographic and healthcare system factors, some factors had a significant association on neonatal mortality.

As it was expected, socioeconomic status had a positive relationship with neonatal health. On other words, neonates of poor families had higher chance of losing their life early. A plethora of studies have already approved of such a relationship; poverty has been shown to be of main determinants of neonatal mortality (21, 25). These findings have been more reported from developing countries. For example, studies from Pakistan, Sudan, Ghana, and Nigeria have shown that low economic status of households is of the salient determinants of neonatal health (20, 26).

Household’s economic status, as a contextual factor, influences neonatal health through having impinges on other factors like mother-related factors, healthcare utilization, and skilled birth attendant.

History of abortion or stillbirth was of main association of neonatal mortality in the present study. This finding was in line with other studies as availability of relevant facilities for abortion and access to them are of factors that affect neonatal mortality (25). For instance, those mothers who already experienced death of an infant (under 1-year-old), had higher chance losing their neonate in future pregnancies (15).

Neonate’s sex, as the only included demographic feature of neonates, had a significant association with neonatal death. This finding accords with other studies (13, 16, 20, 26, 27). In a randomized controlled trial study showed that due to some biological reasons, primary respiratory repression, and low level of consciousness at birth, male neonates experience higher rates of death (28).

Pre-pregnancy and childbirth care factors were other inputs of developed function. Of these factors, skilled birth attendant had a significant association with neonatal health; mothers who had given birth by help of trained personnel had a lower chance of losing their neonate. This finding has been repeatedly reported in other studies (27, 29).

Location of residence and mother education are of factors that had no significant association with neonatal mortality. In rural settings that had no appropriate healthcare services, neonate mortality rate was higher (30). However, considering widespread coverage of healthcare services and PHC in Iran, there is no such a significant difference between rural and urban settings in terms of neonatal mortality in the country. Moreover, with recent scale-up and establishment of national integrated programs, like Integrated Management of Child Illness, Well Baby Care Program, and National Child Mortality Surveillance System, quality and quantity of child healthcare has significantly improved in Iran’s rural settings. Therefore, lack of such a significant difference between rural and urban settings is not unexpected. For mother education level, as factors of economic status, history of abortion or stillbirth, and skilled birth attendant managed to explain a huge part of neonatal mortality, the effect of mother education became insignificant.

Considering notable quota of neonatal mortality from infant and under-5 mortalities, focusing on its reduction will work remarkably towards increase in life expectancy at birth in the country. Findings of present study can also be of high value for policy-makers in order to reduce neonatal mortality. Due attention to poverty elimination and alleviation policies, to mothers’ health across their life course, especially during pregnancy and, development of care services and safe delivery facilities are of ways to increase neonatal health.

One of the main strengths points of the present study is its use of DHS data, as one of the most reliable health-related data, Moreover, use of individual-level data may increase reliability and validity of findings. Most important limitation of the present study was related to input variables. As research team could not access to all neonatal mortality determinants, namely father education and occupation status and history of illness and hospitalization among mothers, the developed model may, for sure, be in need of further development. However, this matter applies almost to all similar studies.

## Conclusion

Economic status of households, history of abortion and stillbirth, neonate’s sex, and skilled birth attendant are of most significant inputs of neonatal health production function in Iran. Resultantly, due attention to economic status of households, provision of appropriate care services for mothers, and improvement of delivery care provided by trained personnel can be of high priority strategies for neonatal health promotion in Iran.

## Ethical considerations

Ethical issues (Including plagiarism, informed consent, misconduct, data fabrication and/or falsification, double publication and/or submission, redundancy, etc.) have been completely observed by the authors.
